# A Allele of *ICAM-1* Rs5498 and *VCAM-1* Rs3181092 is Correlated with Increased Risk for Periodontal Disease

**DOI:** 10.1515/biol-2019-0072

**Published:** 2019-12-31

**Authors:** Qijun Sun, Zongxin Zhang, Yuejian Ou

**Affiliations:** 1 Stomatology Therapeutic Center, Huzhou Central Hospital, Affiliated Cent Hosp Huzhou University, Huzhou 313000, P.R. China; 2 Clinical Laboratory, Huzhou Central Hospital, Affiliated Cent Hosp Huzhou University, Huzhou 313000, P.R. China

**Keywords:** Periodontal disease, *ICAM-1* gene, *VCAM-1* gene, Polymorphism, Logistic regression, Clinical index, Denaturing high performance liquid chromatography, Protein expression

## Abstract

**Objective:**

Periodontal disease (PD) is viewed today as multifactorial problems initiated and sustained by bacteria but significantly modified by the body’s response to bacterial plaque. Recent studies have suggested that gene polymorphisms could be involved in the pathophysiology of periodontitis. This study aimed to investigate a possible correlation of the polymorphisms of intercellular adhesion molecule-1 (*ICAM-1*) and vascular cell adhesion molecule-1 (*VCAM-1*) with PD.

**Methods:**

The genotypes of *ICAM-1* and *VCAM-1* were initially determined in PD patients using denaturing high performance liquid chromatography (DHPLC). ELISA was then conducted to measure *ICAM-1* and *VCAM-1* protein levels. Next, the association of *ICAM-1/VCAM-1* genotype distribution and expression with clinical indicators and severity of PD was analyzed.

**Results:**

PD patients contained increased levels of hemoglobin A1c (HbA1c), total cholesterol (TC), triglyceride (TG), and low-density lipoprotein (LDL), increased *ICAM-1* and *VCAM-1* protein levels, and decreased high-density lipoprotein (HDL) level. The GG genotype and G allele at *ICAM-1* rs5498, as well as the AG and GG genotypes and G allele at *VCAM-1* rs3181092 may reduce PD risk.

**Conclusion:**

To sum up, the overexpressed *ICAM-1* and *VCA M-1* as well as A allele of *ICAM-1* rs5498 and *VCAM-1* rs3181092 is associated with the onset of PD.

## 1 Introduction

Periodontal disease (PD) is a chronic infectious disease that occurs in periodontal soft tissues and supporting structures [1]. The main clinical manifestations of PD include gingival infection and inflammation, formation of periodontal pockets, and detachment of loose teeth [2, 3, 4, 5, 6]. A previous study has reported that PD affects four out of every five adults [6]. The factors responsible for PD usually include gene polymorphisms, plaque microorganisms, bad habits, mental pressure and aging [6, 7, 8, 9]. Further researches on the etiology of PD may help an early diagnosis and effective treatment of PD.

Recent studies have confirmed that PD is closely related to the regulation of inflammation factors [10, 11, 12], including inflammatory cytokines such as IL-1 and IL-6 [13, 14, 15], acute phase proteins such as C-reactive protein (CRP) [16], and coagulation proteins such as intercellular adhesion molecule-1 (*ICAM-1*) and vascular cell adhesion molecule-1 (*VCAM-1*) [17, 18, 19]. A study found that in the development of PD, a higher level of Porphyromonas gingivalis (*P. gingivalis*) microorganisms appears in the body and guides the expression of *ICAM-1* and *VCAM-1* genes, resulting in intravascular metabolism imbalance [20]. This suggests that *ICAM-1* and *VCAM-1* are possibly the important factors in the pathogenesis of PD. *ICAM*-1 and *VCAM*-1 belong to the immunoglobulin superfamily of endothelial adhesion molecules [21]. Accumulating evidence has revealed that *ICAM-1* and *VCAM-1* polymorphisms correlate to myocardial infarction and coronary artery disease [22, 23, 24]. Because the pathophysiology of PD is similar to that of these diseases, which is inflammatory, and there is still no sufficient data on genetic polymorphisms of *ICAM-1* and *VCAM-1* genes at present [25], this study will focus on the polymorphisms of *ICAM-1* and *VCAM-1* genes, and compare the genotypes of *ICAM-1* and *VCAM-1* between healthy people and PD patients. By doing so, it is hoped to discover the gene polymorphisms closely related with PD.

## 2 Materials and methods

### 2.1 Research subjects

Chronic PD patients were enrolled from outpatient department of stomatology at the Huzhou Central Hospital, Affiliated Cent Hosp Huzhou University between August 2012 and August 2016. PD was diagnosed according to the Armitage standard (1999) [26]. The inclusion criteria were as follows: 1, patients with at least 14 teeth for periodontal evaluation (including at least 4 molar teeth); 2, patients without kin relationship; 3, patients without systemic diseases; 4, patients without history of orthodontic treatment; 5, patients with no use of antibiotics 3 months before the experiment. The exclusion criteria were as follows: 1. the patients received systematic PD treatment; 2. the patients received teeth cleanse treatments 1 year before the experiment; 3. women were in pregnancy and breast-feeding period. The patients in the control group were healthy individuals who were admitted to the outpatient department of stomatology at the Huzhou Central Hospital, Affiliated Cent Hosp Huzhou University during the same period of time. The individuals in the control group had a full dentition periodontal probing depth of ≤ 3 mm, no attachment loss, gingival recession of ≤ 1 mm, and no teeth bleeding during the dental probing.

**Informed consent**: Informed consent has been obtained from all individuals included in this study.

**Ethical approval**: The research related to human use has been complied with all the relevant national regulations, institutional policies and in accordance the tenets of the Helsinki Declaration, and has been approved and supervised by the ethics committee at Huzhou Central Hospital.

### 2.2 Denaturing high performance liquid chromatography (DHPLC) [27, 28]

A total of 5 mL peripheral blood was acquired from each patient with empty stomach in the morning. The blood samples were anti-coagulated by ethylenediamine tetraacetic acid (EDTA) according to the instructions of a DNA Kit (Qiagen Company, Hilden, Germany). The DNA concentration in the samples was measured. The absorbance at 260 nm and 280 nm was measured, and their ratio was always between 1.8 and 2. The reverse transcription quantitative polymerase chain reaction (RT-qPCR) amplification primers for the testing sites were designed and verified using the Primer Premier 5.0 software, and primers were synthesized by Shanghai Sangon Biotechnology Co., Ltd. (Shanghai, China). The primer sequences were: CCATCGGGGAATCAGTGACT (forward sequence of rs5498) and ATGACTGCGGCTGCTACCA (reverse sequence of rs5498), GGAGATGTTTCACGAAGTTTGT (forward sequence of rs3181092) and AAGGGTCACATTTTGAGTATCC (reverse sequence of rs3181092). The RT-qPCR reaction conditions were as follows: pre-denaturation at 94oC for 2 min, 35 cycles of denaturation at 94oC for 1 min, annealing at 52oC for 1 min and extension at 72oC for 1 min, and a final elongation step at 72oC for 5 min. The RT-qPCR products were stored at 4oC and all of the genotypes were detected by DHPLC under the conditions of partial denaturation. During DHPLC, the column temperature was 59.3oC, and the flow rate of mobile phase was 0.9 mL/min. The genotyping of fragment length gene at each site was accomplished in two steps: in the first step, samples with bimodal DHPLC curves were classified as heterozygous genotype; in the second step, the RT-qPCR samples producing unimodal curves were equally mixed with samples with homozygous genotypes confirmed by sequencing, and then measured by DHPLC again. The samples with unimodal curves were classified as wild-type homozygote, while the samples with bimodal curves were classified as homozygous mutates, and the mutation was further confirmed by gene sequencing.

### 2.3 Determination of periodontal index and the severity of illness in PD patients

Plaque index (PI) was measured using the bacteria plaque index and scoring method of Silness and Loe [29]: 0 = no bacterial plague in gingival zone; 1 = thin bacterial plaques on the surface of gingival zone, which cannot be observed directly by visual examination and can be scraped out with a dental probe; 2 = moderate amount of bacterial plaques visible in gingival zone or on adjacent surfaces; 3 = large amount of soft fouling in gingival sulcus, gingival zone and adjacent areas. Bleeding index (BI) was determined using the bleeding indexing method proposed by Mazza *et al*. (1981) [30], and was recorded in 6 levels. Probing depth was used to measure the distance from the bottom of gingival pockets to the upper edge of the gingival zone, and 6 loci were measured for each tooth in mm units. Clinical attachment level (CAL) was defined as the value of probing depth minus the distance from the cementoenamel junction to the gingival zone. In case of gingival recession, CAL was the value of probing depth plus the distance of gingival recession.

The Centers for Disease Control and Prevention (CDC) of USA and the American academy of periodontology (AAP) provide grading standards for periodontitis based on the PD surveillance data from different populations [31]: mild periodontitis: < 4 mm periodontal pockets and 1 to 2 mm CAL, alveolar bone adsorption less than one third of root length as detected by X-ray; moderate periodontitis: ≥ 4 mm CAL at ≥ 2 interproximal sites from different teeth, or ≥ 5 mm periodontal pockets at ≥ 2 interproximal sites from different teeth; severe periodontitis: ≥ 6 mm CAL at ≥ 2 interproximal sites from different teeth, or ≥ 5 mm periodontal pockets at ≥ 1 interproximal sites from different teeth.

### 2.4 Double-antibody-sandwich ABC-enzyme-linked immunosorbent assay (ELISA)

The blood samples were centrifuged at 4oC for 10 min at 3000 rpm/min, and the plasma aliquots were separated and transferred into centrifuge tubes according to the instructions of the Kit (ICAM-1 antibody [Abcam Inc., Cambridge, MA, USA, ab174445] [32] and VCAM-1 antibody [Abcam Inc., Cambridge, MA, USA, ab46118] [33]). The serum samples to be tested were diluted, added into enzyme coated plates, and incubated at 37oC for 30 min. Each well was added with 50 μL of ELISA reactive solution, and incubated at 37oC for 30 min. Subsequently, each well was added with 50 μL of chromogenic agent A and 50 μL of chromogenic agent B, mixed by shaking for 30 s, and colored at 37oC in the dark for 15 min. The optical density (OD) values of each well were measured at a wavelength of 450 nm. Standard curves were then drawn with OD value as abscissa and concentration as vertical coordinate. The concentrations were 25, 50, 100, 200 and 400 ng/L.

### 2.5 Statistical methods

Statistical analysis was carried out using SPSS 21.0 software (IBM Corp. Armonk, NY, USA). Measurement data were presented as mean ± standard deviations (s.d.). Data obeying normal distribution and homogeneity of variance between two groups were compared using independent sample t-test. Data not conforming homogeneity of variance between two groups were compared using Welch’s approximate t-test. Mann-Whitney U (non-parametric) test was used for data with skewed distribution. Count data were presented using rate or percentage. Pearson’s chi-square test is used for comparing inter-group frequencies when N ≥ 40 and the expected value of all cells T ≥ 5, and chi-square test with correction for continuity was used when N ≥ 40 and 1 ≤ T ≤ 5. Multivariate analysis was done using a binary logistic regression analysis. Chi-square tests were used to verify whether the genotypes were consistent with the Hardy-Weinberg equilibrium model. The *p* meant bilateral probability, and a *p* value < 0.05 was indicative to be statistically significant.

## 3 Results

### 3.1 PD correlates to increased ICAM-1 and VCAM-1 levels

There were 196 chronic PD cases, with 102 males and 94 females at a mean age of 45.30 ± 7.20 years included in our study. There were 192 cases in the control group, with 92 males and 100 females at a mean age of 44.89 ± 5.80 years. Following demographic characteristic analyses in the two groups (Table 1), no statistical difference was detected in the gender, age, smoking and drinking history between the two groups (all *p* > 0.05). The levels of hemoglobin A1c (HbA1c), serum total cholesterol (TC), triglyceride (TG) and low-density lipoprotein (LDL) in the PD group were much higher than those in the control group, while that of high-density lipoprotein (HDL) in the PD group was much lower than that in the control group (all *p* < 0.05). In addition, ELISA showed that the ICAM-1 level in the PD group was 723.60 ± 158.02 ng/mL, which was higher than that that observed in the control group (589.96 ± 88.95 ng/mL) (*p* = 0.001). VCAM-1 level in the PD group was 1226 ± 201 ng/mL, higher than that observed in the control group (957.98 ± 192 ng/mL) (*p* = 0.001).

**Table 1:** ELISA reveals that PD is associated with increased ICAM-1 and VCAM-1 levels

Parameters	PD (n = 196)	Control (n = 192)	c^2^/t/Z	*p*
Gender (Male/Female)	102/94	92/100	0.660	0.417
Average Age	45.30 ± 7.20	44.89 ± 5.80	-0.671	0.502
Smoking (Y/N)	72/124	60/132	1.300	0.254
Alcohol (Y/N)	82/114	68/124	1.686	0.194
HbAlc (%)	5.84 ± 1.43	5.26 ± 0.75	3.268	0.001
TC (mmol/L)	3.91 ± 0.78	3.72 ± 0.49	2.866	0.004
TG (mmol/L)	1.37 ± 0.41	1.24 ± 0.33	3.436	0.001
LDL (mmol/L)	3.62 ± 0.78	3.35 ± 0.58	3.863	0.001
HDL (mmol/L)	1.09 ± 0.38	1.21 ± 0.39	3.070	0.002
SICAM-1(ng/mL)	723.60 ± 158.02	589.96 ± 88.95	13.230	0.001
SVCAM-1(ng/mL)	1213.73 ± 242.19	957.98 ± 192	13.430	0.001

Notes: Y = Yes; N = No; HbA1c = hemoglobin A1c; TC = total cholesterol; TG = triglyceride; LDL = low-density lipoprotein; HDL = high-density lipoprotein; PD = periodontal disease; ELISA = enzyme-linked immunosorbent assay.

### 3.2 A alleles of rs5498 in ICAM-1 and rs3181092 in VCAM-1 increase the risk of PD

In the control group, the Hardy-Weinberg equilibrium was verified with a goodness of fit test. The results showed that the genotype distributions at rs5498 in *ICAM-1* gene and rs3181092 in *VCAM-1* gene were consistent with the Hardy-Weinberg equilibrium (*p* > 0.05). Therefore, the samples in this study were random samples and were representative of the general population.

The genotypes and allele frequency distributions at rs5498 in *ICAM-1* gene and rs3181092 in *VCAM-1* gene are shown in Table 2. The genotypes and allele distributions at rs5498 in *ICAM-1* gene were different between the PD group and the control group (*p* < 0.05). The GG genotype may reduce PD risk (odds Ratio (OR) = 0.529, 95% confidence intervals (CI) = 0.292 to 0.959, *p* = 0.035), and the G allele may also reduce PD risk (OR = 0.721, 95% CI = 0.539 to 0.963, *p* = 0.026). At rs3181092 of *VCAM-1* gene, the AG genotype (OR = 0.561, 95% CI = 0.348 to 0.905, *p* = 0.017), GG genotype (OR = 0.263, 95% CI = 0.143 to 0.481, *p* = 0.001) and G allele (OR = 0.530, 95% CI = 0.398 to 0.705, *p* = 0.001) may all decrease the risk of PD.

**Table 2:** A alleles at rs5498 of *ICAM-1* and rs3181092 of *VCAM-1* is associated with increased risk for PD: n (%)

Genotype	PD group	Control group	*p*	OR value (95% CI)
			
	(n = 98)	(n = 96)		
5498A/G				
AA	84 (42.86)	65 (33.85)	Ref.	
AG	86 (43.88)	89 (46.35)	0.193	1.337 (0.862 - 2.075)
GG	26 (13.26)	38 (19.80)	0.035	0.529 (0.292 - 0.959)
AG + GG	112 (57.13)	127 (66.15)	0.068	0.682 (0.452 - 1.030)
A allele	254 (64.80)	219 (57.03)	Ref.	
G allele	138 (35.20)	165 (42.97)	0.026	0.721 (0.539 - 0.963)
3181092A/G				
AA	72 (36.73)	40 (20.83)	Ref.	
AG	98 (50.00)	97 (50.52)	0.017	0.561 (0.348 - 0.905)
GG	26 (13.27)	55 (28.65)	0.001	0.263 (0.143 - 0.481)
AG + GG	124 (63.27)	152 (79.17)	0.001	0.453 (0.288 - 0.713)
A allele	242 (61.73)	177 (46.09)	Ref.	
G allele	150 (38.27)	207 (53.91)	0.001	0.530 (0.398 - 0.705)

Notes: OR = odds ratio; CI = confidence intervals; *ICAM-1* = intercellular adhesion molecule-1; *VCAM-1 =* vascular cell adhesion molecule-1; PD = periodontal disease.

### 3.3 AA genotypes of rs5498 and rs3181092 of ICAM-1 and VCAM-1 genes correlate to the increase of BI index, CAL and the severity of PD

The correlation of rs5498 and rs3181092 of *ICAM-1* and *VCAM-1* genes with clinical features of PD is shown in Table 3. The genotype distribution at rs5498 of *ICAM-1* gene and rs3181092 of *VCAM-1* gene was related to the BI index of PD patients: compared with patients with AA genotype, patients with AG + GG genotypes exhibited decreased BI index (all *p* < 0.05), CAL: compared with patients with AA genotype, patients with AG + GG genotypes exhibited diminished CAL (all *p* < 0.05) and the severity of PD: severe patients with AA genotype at rs5498 of *ICAM-1* accounted for 48.81%, while mild patients with AG + GG genotypes accounted for 56.25% (all *p* < 0.05); severe patients with AA genotype at rs3181092 of *VCAM-1* accounted for 38.89%, while severe patients with AG + GG genotypes accounted for 21.77%, which was lower than severe patients with AA genotype (all *p* < 0.05).

**Table 3:** AA genotypes of rs5498 and rs3181092 of ICAM-1 and VCAM-1 genes is associated with increase of BI index, CAL and the severity of PD

Clinical features	rs5498		*P*	rs3181092		*P*
	AA	AG + GG		AA	AG + GG	
PI	0.97 ± 0.15	0.94 ± 0.16	0.183	0.965 ± 0.15	0.95 ± 0.16	0.667
BI	3.18 ± 0.55	2.83 ± 0.40	0.001	3.11 ± 0.54	2.91 ± 0.47	0.007
Probing depth (mm)	2.88 ± 0.36	2.85 ± 0.28	0.512	2.83 ± 0.38	2.82 ± 0.28	0.833
CAL (mm)	3.15 ± 0.84	2.85 ± 1.04	0.001	3.85 ± 0.33	2.48 ± 0.86	0.001
Severity						
Mild	18 (21.43)	63 (56.25)		27 (37.50)	54 (43.55)	
Medium	25 (29.76)	35 (31.25)	0.001	17 (23.61)	43 (34.68)	0.030
Severe	41 (48.81)	14 (12.50)		28 (38.89)	27 (21.77)	

Notes: PI = plaque index; BI = bleeding index; CAL = clinical attachment level; PD = periodontal disease; *ICAM-1* = intercellular adhesion molecule-1; *VCAM-1 =* vascular cell adhesion molecule-1.

### 3.4 AA genotype of rs5498 and rs3181092 increases levels of *SICAM-1* and *SVCAM-1*

The ELISA results (Table 4) showed that compared with the patients with AA genotype of rs5498, patients with AG + GG genotypes presented with decreased *SICAM-1* and *SVCAM-1* levels (all *p* < 0.05). Compared with the patients with AA genotype of rs3181092, there was a downward trend in *SICAM-1* and *SVCAM-1* levels in patients with AG + GG genotypes (all *p* < 0.05).

**Table 4:** ELISA shows that AA genotype of rs5498 and rs3181092 increases SICAM-1 and SVCAM-1 levels

		rs5498			rs3181092	
	
	AA	AG + GG	*p*	AA	AG + GG	*p*
SICAM-1 (ng/mL)	841.69 ± 151.17	635.03 ± 91.16	0.001	762.46 ± 184.82	720.11 ± 106.00	0.008
SVCAM-1 (ng/mL)	1405.21 ± 163.23	1070.12 ± 185.91	0.001	1262.83 ± 246.12	1185.22 ± 236.21	0.030

Notes: *ICAM-1* = intercellular adhesion molecule-1; *VCAM-1 =* vascular cell adhesion molecule-1; ELISA = enzyme-linked immunosorbent assay.

### 3.5 HbA1c, LDL and AA genotype are risk factors for PD

The related risk factors of PD were analyzed using the logistic regression analysis. The results revealed that HbA1c (Exp (B) = 1.512, 95% CI = 1.185 to 1.930, *p* = 0.001) and LDL (Exp (B) = 1.783, 95% CI = 1.204 to 2.641, *p* = 0.004) may amplify PD risk, while HDL may reduce the risk of PD (Exp (B) = 0.418, 95% CI = 0.208 to 0.840, *p* = 0.014).

Using the AA genotype at rs3181092 in *VCAM-1* gene as a reference, the genotype AG + GG may abate the risk of PD (Exp (B) = 0.344, 95% CI = 0.127 to 0.932, *p* = 0.036) (Table 5).

**Table 5:** Logistic regression analysis demonstrates that HbA1c, LDL and AA genotype are risk factors for PD

Factors	B	S.E.	Wald	df	Sig.	Exp(B)	95% CI
HbA1c	0.414	0.414	11.072	1	0.001	1.512	1.185 - 1.930
TC	0.219	0.221	0.978	1	0.323	1.245	0.807 - 1.921
TG	1.145	0.374	9.352	1	0.002	3.142	1.508 - 6.543
LDL	0.578	0.200	8.336	1	0.004	1.783	1.204 - 2.64
HDL	-0.873	0.357	5.998	1	0.014	0.418	0.208 - 0.840
S*ICAM-1*	0.005	0.002	12.425	1	0.001	1.005	1.002 - 1.008
S*VCAM-1*	0.004	0.001	22.570	1	0.001	1.004	1.002 - 1.005
rs5498	0.601	0.475	1.599	1	0.206	1.823	0.719 - 4.623
rs3181092	-1.066	0.508	4.404	1	0.036	0.344	0.127 - 0.932

Notes: HbA1c = hemoglobin A1c; TC = total cholesterol; TG = triglyceride; LDL = low-density lipoprotein; HDL = high-density lipoprotein; CI = confidence intervals; df = degree of freedom; *ICAM-1* = intercellular adhesion molecule-1; *VCAM-1 =* vascular cell adhesion molecule-1; PD = periodontal disease.

## 4 Discussion

The aim of this study was to investigate the relationship between *ICAM-1* and *VCAM-1* gene polymorphisms and the progression of PD. Collectively, this study confirmed that the polymorphisms of *ICAM-1* and *VCAM-1* genes and their protein levels were related to PD progression. Therefore, the polymorphisms of *ICAM-1* and *VCAM-1* genes may be used as clinical diagnostic references. At the meantime, the levels of other factors such as HbA1c, TC, TG, HDL and LDL are also proved to be associated with PD.

*ICAM-1* and *VCAM-1* act as adhesion molecules and generate a variety of cell adhesion effects with many types of cells [34, 35]. The balance of ICAM-1 and VCAM-1 expression might be important for the regulation of leucocytes infiltration and retention in periodontally diseased tissues [36]. An adequate amount of expression can promote immune responses in the body, while an excessive expression hinders the immune system and inhibits the cleansing of exogenous antigens and harmful substances from the body [37]. For normal people, the inner epidermal cells distribute uniformly across the surface of teeth, and macrophages can produce non-specific immune responses against harmful microorganisms, which are subsequently degraded and digested to prevent damage [38, 39]. However, *ICAM-1* and *VCAM-1* were highly expressed inside the gum tissue of PD patients, thereby inducing endothelial cells to produce excessive amounts of adhesion molecules and to form a barrier around the teeth [40]. In agreement with our findings, ICAM-1 and VCAM-1 are inflammatory markers, the overmuch of which has been found to amplify the risk of PD [41]. In this situation, the macrophages cannot attach to exogenous microorganisms in order to digest them, resulting in excessive proliferation of microbes and tooth damage [42]. The results from this study showed that the expression of *ICAM-1* and *VCAM-1* was higher in the plasma of PD patients, and this conclusion was consistent with a previous study [43]. In addition, the experimental results of this study proved the association between PD and the polymorphism at rs5498 of *ICAM-1* and rs3181092 of *VCAM-1* genes, respectively. According to the NCBI website (http://www.ncbi.nlm.nih.gov/projects/SNP/), rs5498 of *ICAM-1* gene is located at position 1724 in the coding sequence, which may lead to mis-sense mutation. Similarly, rs3181092 of *VCAM-1* gene is located in the non-coding sequence subsequent to the exon 9. In general, mis-sense mutations directly affect cell functions [44]. Another key observation from the study was that such mutations in *ICAM-1* and *VCAM-1* genes may help to decrease the risk of PD. A previous study revealed that *ICAM-1* rs5498 and *VCAM-1* rs1041163 polymorphisms lead to chronic periodontitis [25]. A higher prevalence of G allele carriers (AG + GG) in patients was found in coronary artery disease (CAD) patients as compared to controls, and in addition, ICAM1 rs5498 polymorphism increases the risk for CAD [45], where periodontal destruction drives CAD severity [46]. It may be possible that the function of forming shielding barriers by mutated *ICAM-1* is relatively weakened, and hence the macrophages can properly generate non-specific immunity in this situation. It has been shown that non-coding sequences are often related with gene expression [47]. Therefore, *VCAM-1* mutation may reduce its expression and help to reduce the formation of shielding barriers on tooth surface.

Other risk factors such as HbA1c, serum TC, TG, HDL and LDL levels were also determined in this study. Among them, the levels of HbA1c, serum TC, TG and LDL were significantly increased in PD patients, while the HDL expression in PD patients was relatively lower. Previous studies have shown that PD is often positively related with obesity or diabetes [48, 49]. The aforementioned risk factors are often used as index factors to determine body fat content and cardiovascular disease, and their changes in PD are similar to those observed in patients with obesity and diabetes [50, 51]. An environment with high blood lipids and glucose is the most important cause of inflammatory responses in the body [52], which may trigger the expression of relevant factors such as IL-6, IL-10 and TNF-α, thereby increasing the risk of periodontal inflammation [53]. Moreover, HbA1c is demonstrated to be a predictor for diabetes risk in patients with chronic periodontitis since the HbA1c average in healthy controls is significantly higher than those affected with severe chronic periodontitis [54]. Oxidized LDL elicits gingival epithelial cell inflammatory responses through an activation of the NF-κB pathway [55]. Such observation may be noteworthy in the diagnosis and treatment of PD patients.

However, there were still some limitations about this study: first of all, the number of patients in this study was relatively small, and the results might have certain deviations and limitations from the statistics’ point of view. Secondly, this study mainly concentrated on the role of *ICAM-1* and *VCAM-1* gene polymorphisms in PD; nevertheless, there are other factors including environmental effects and health habits that may also affect PD. These factors were not studied thoroughly in this research, and may need further investigations in order to obtain a more comprehensive conclusion.

## 5 Conclusion

In this research, it is proved that polymorphisms and protein levels of *ICAM-1* and *VCAM-1* genes may be related to PD and affect its progression. Such findings may have certain reference values for future clinical diagnosis and treatment of PD.

## Abbreviations

ICAM-1: intercellular adhesion molecule-1
VCAM-1: vascular cell adhesion molecule-1
PD: periodontal disease
DHPLC: denaturing high performance liquid chromatography
HbA1c: hemoglobin A1c
TC: total cholesterol
TG: triglyceride
LDL: low-density lipoprotein
HDL: high-density lipoprotein
CRP: C-reactive protein
DHPLC: Denaturing high performance liquid chromatography
EDTA: ethylenediamine tetraacetic acid
PI: plaque index
BI: bleeding index
CAL: clinical attachment level
CDC: Centers for Disease Control and Prevention
AAP: american academy of periodontology
ELISA: enzyme-linked immunosorbent assay
OD: optical density
RT-qPCR: reverse transcription quantitative polymerase chain reaction
s.d.: standard deviations
CI: confidence intervals
